# Microbiota Effect on Trimethylamine N-Oxide Production: From Cancer to Fitness—A Practical Preventing Recommendation and Therapies

**DOI:** 10.3390/nu15030563

**Published:** 2023-01-21

**Authors:** Edoardo Tacconi, Giuseppe Palma, Davide De Biase, Antonio Luciano, Massimiliano Barbieri, Filomena de Nigris, Francesca Bruzzese

**Affiliations:** 1 Department of Human Science and Quality of Life Promotion, San Raffaele Roma Open University, 00166 Rome, Italy; 2 S.S.D. Sperimentazione Animale, Istituto Nazionale Tumori-IRCCS-Fondazione G. Pascale, 80131 Naples, Italy; 3 Department of Pharmacy, University of Salerno, Via Giovanni Paolo II 132, 84084 Fisciano, Italy; 4 Department of Precision Medicine, School of Medicine, Università degli Studi della Campania “Luigi Vanvitelli”, Via De Crecchio 7, 80138 Naples, Italy

**Keywords:** trimethylamine N-oxide (TMAO), trimethylamine (TMA), gut microbiota, diet, choline, L-carnitine, gastrointestinal cancer, colorectal cancer

## Abstract

Trimethylamine N-oxide (TMAO) is a microbial metabolite derived from nutrients, such as choline, L-carnitine, ergothioneine and betaine. Recently, it has come under the spotlight for its close interactions with gut microbiota and implications for gastrointestinal cancers, cardiovascular disease, and systemic inflammation. The culprits in the origin of these pathologies may be food sources, in particular, high fat meat, offal, egg yolk, whole dairy products, and fatty fish, but intercalated between these food sources and the production of pro-inflammatory TMAO, the composition of gut microbiota plays an important role in modulating this process. The aim of this review is to explain how the gut microbiota interacts with the conversion of specific compounds into TMA and its oxidation to TMAO. We will first cover the correlation between TMAO and various pathologies such as dysbiosis, then focus on cardiovascular disease, with a particular emphasis on pro-atherogenic factors, and then on systemic inflammation and gastrointestinal cancers. Finally, we will discuss primary prevention and therapies that are or may become possible. Possible treatments include modulation of the gut microbiota species with diets, physical activity and supplements, and administration of drugs, such as metformin and aspirin.

## 1. Introduction

The interest in human microbiota and its modulation of interactions between food sources and some pathologies such as the metabolic syndrome, cardiovascular disease and some types of cancer has been growing in the scientific world. Even if there is a long way to go, our attention has been directed to a molecule involved in systemic inflammation, called trimethylamine N-Oxide (TMAO) [1,2,3]. TMAO is an amino oxide, produced from the trimethylamine (TMA) through oxidation by some liver enzymes called flavin monooxygenases 1 and 3 (FMO1 and FMO3). Three forms of the enzyme, FMO1 found in fetal liver, FMO3 found in adult liver, and genes clustered in the 1q23-q25 region encode FMO3. Flavin-containing monooxygenases are NADPH-dependent flavoenzymes that catalyze the oxidation of soft nucleophilic heteroatom centers in xenobiotics, such as pesticides and drugs. The human FMO3 enzyme catalyzes several types of reactions, including the N-oxygenation of primary, secondary, and tertiary amines [4]. TMA production is indirectly influenced by some specific compounds such as L-carnitine, choline and other isoforms, betaine, and lecithin, and directly from gamma-butyrobetaine [2,5,6]. These compounds are metabolized in the gut through interactions with some of microbiota bacteria by different enzymes. First, the major clusters of TMA production start from the mouth with *Streptococcus sanguis* and the genes CutC and CutD, that are necessary for *Desulfovibrio* and *Desulfovibrio desulfuricans* to convert choline in TMA [7]. Other genes such as CntA and CntB contained in *Actinobacter* and *Serratia*, promote the oxidoreductase enzymes from L-carnitine to TMA. Furthermore, YeaW and YeaX are involved in oxygenase and oxidoreductase enzymes for choline, betaine, L-carnitine and gamma-butyrobetaine. Bacteria from gamma-proteobacteria like *Escherichia coli*, *Citrobacter*, *Klebsiella pneaumoniae*, and *Shigella*, *Achromobacter* from the strain of *Betaproteobacteria*, *Sporosarcina* from *Firmicutes*, and *Actinobacteria* have orthologue and homologue enzymes such as CntA, CntB, YeaW and YeaX, which encode the gene that can convert all food compounds including choline, betaine, lecithin, gamma-butyrobetaine, ergothioneine and L-carnitine into TMA [7].

Choline, choline esters (e.g., phosphatidylcholine) and lecithin, all important and essential nutrients for the nervous system health [8,9], are converted in TMA by TMA lyase; also, choline can be oxidase in betaine by choline dehydrogenase and choline aldehyde dehydrogenase, which is converted to TMA by betaine oxidoreductase [10]. Furthermore, lecithin, which is a source of choline, can be re-converted to choline by phospholipase D and again converted to TMA [9].

L-carnitine is oxidized directly to TMA by the carnitine oxidoreductase, and indirectly from gamma-butyrobetaine [11]. L-carnitine can be converted by gamma-butyrobetaine hydroxylase in gamma-butyrobetaine, and eventually in TMA by TMA lyase [12]. When the conversion from TMA to TMAO is inhibited, it manifests a particular metabolic syndrome called trimethylaminuria, or “*fish odor syndrome*”, due to the accumulation of TMA molecules excreted in the urine, sweat and breath which smell like putrid fish [13]. This pathology occurs with a mutation in the gene encoding the liver enzyme FMO3 [14], single nucleotide polymorphism E158K and E308G, which has 10-fold higher specific activity to convert TMA in TMAO then the FMO1 [15]. In some patients, low choline food sources have been recommended [16], but a promising future therapy is to intervene on gut microbiota to modulate the production of TMA and TMAO [17]. Strong evidence correlates high levels of L-carnitine with high levels of TMAO and its potential pro-atherogenic role [18,19] and its role was confirmed by a meta-analysis showing that all causes of mortality increased by 7% per each 10 µmol/L increment of circulating TMAO [20]. Several studies reported a remarkable increment of TMAO achieved by supplementation of L-carnitine, but some of them observed alterations of cardiovascular disease (CVD) markers [21,22,23,24,25]. A diet rich in animal protein, those with high fat content, such as processed and unprocessed meats [26], containing compounds such as choline, carnitine, but even betaine and lecithin in plants usually consumed [27], can produce molecules of TMA by microbiota interaction which can be eventually converted in TMAO [28]. Another molecule in meats, from liver and kidneys, mushrooms, and several type of beans, which is directly involved in TMA production, is ergothioneine, which is converted in TMA through ergothionase enzyme [10]. The TMA molecules are produced directly or indirectly mainly from food compounds containing L-carnitine and choline, but also from betaine and lecithin, ergothioneine and gamma-butyrobetaine. TMAO production is not a clear consequence but can potentially be influenced by dysbiosis and individual polymorphisms in the expression of FMO3 in the liver [14]; as well as the intake of fish and other seafood in the diet [26], and a more systemic disease [29,30]. As well as genetic factors and environmental factors, recent evidence suggests that even the metabolites deriving from the microbiota can play a protective role or promote the onset of tumors. These bacteria produce toxic metabolites, such as secondary bile salt from primary bile salt, hydrogen sulfide, trimethylamine-N-oxide (TMAO) from choline, indoxyl sulfate from amino acid tryptophan, and many more which are likely to promote inflammation, and prolonged inflammation can develop into cancer [5,7,31,32]. The potential role of several gut bacteria metabolites may cause localized inflammation in normal tissue of colon and promote the genotoxicity of intestinal epithelial cells, determining dysplasia and finally, colorectal cancer (CRC) [1,33].

## 2. TMAO in Physiological Conditions

TMAO molecules are produced in the liver, enter in the blood stream, from where most of them are excreted with urine within 24 h [7], and some can be reconverted in TMA by TMAO reductase [29]. In the intestine, the TMA conversion starts from choline and its isoform with a specific glycyl-radical-enzyme (GRE), GRE choline TMA-lyase (cutC) and its precursor GRE activase (cutD) [34] and from L-carnitine and gamma-butyrobetaine with Rieske-type oxygenase/reductase (cntA/B) [11]. The production of TMAO mainly depends on its pre-substrate TMA and the expression of liver enzymes FMO1 and mostly FMO3 [28]. TMAO levels also depend on genetic factors such as the presence of the E158K and E308G polymorphism on FMO3 [14]; another gene, founded in mice, called Slc30a7, associated with a zinc transporter (ZNT7) [35], seems to be correlated with plasma TMAO levels [36]. Although, as some studies report, the genetic factors in human associated with TMAO plasma levels are more complex [37] and correlated with pathological environment, such as CVD and comorbidities [38,39,40]. Some food sources have free TMA molecules that are absorbed by enterocytes by passive diffusion, oxidized, and expelled with a 3:95 TMA:TMAO ratio through urine (95%), feces (4%) and breathing (1%) [7]. The FMO3 enzyme is expressed in lungs, adrenals, and aorta. There is a gender difference in TMA activity, which is greater in female than male rats [41]. In males, the predominant steroid, testosterone, is responsible for a lower expression of FMO3 in the liver, whereas high levels of estrogen seem to elevate it [41], but a cohort study underlines that males have more TMAO levels than females [42]. However, some studies do not confirm a hormonal influence nor detect any significant sex differences in the levels of circulating TMAO [43,44,45]. Many other factors potentially mediate the production of TMAO from TMA, e.g., age, pathological status (CVD, low-grade inflammation, diabetes, tumors, genetic polymorphisms, protein-specific transport; sedentary life and inadequate nutritional status that may lead to gut dysbiosis. The production of TMA in physiological condition occurs through the interactions of compounds and some strains of gut microbiota. An in vitro study observed that the production was mainly by *Firmicutes* and *Proteobacteria* phyla, but not *Bacteroidetes* [46]. Yet other studies reported that all the production of TMA from choline and L-carnitine resulted from *Firmicutes*, *Proteobacteria* phyla, and none from *Bacteroidetes* [34,47]. One week with an antibiotic treatment to suppress some strains in the human gut leads to a reduction of TMAO levels, even with an L-carnitine [30] and choline supplementation [48]. From these studies, one could conclude that the amount of TMAO circulating is not only modulated by the ingestion of choline and L-carnitine food sources, but also by gut strains, in particular “high TMAO producers” with a high *Firmicutes/Bacteroidetes* ratio (roughly 2:1) [49]. In some studies, it was reported that a healthy gut microbiota in the adult is the one that has a high *Firmicutes* percentage on *Bacteroidetes* or roughly equal relative percentage [50,51]. Sometimes in other studies, it was found that obese people have a higher level of *Bacteroidetes* than thin individuals and a decrease of this strain has been seen after a period of caloric restriction [52]. Other gut microbes who involved in the TMA production are *Deferribacteraceae*, *Anaeroplasmataceae*, *Prevotellaceae* (*Bacteroidetes* phylum abundant in subjects consuming mostly starchy carbohydrates and fiber [53]), and *Enterobacteraceae* [34,54]. Furthermore, an increased TMAO has been found in those who consume a large amount of seafood per day. Similar levels were also found after the consumption of fish rich in omega-3 polyunsaturated fatty acids (e.g., salmon) [55], which may be due to their high content of free TMAO molecules [56]. Half of free TMAO intake from food is absorbed and eliminated in urine, the other half may be converted in TMA by the enzyme TMAO reductase [10]. Whether TMAO levels are correlated with milk and dairy products remains controversial [44]. Another interesting point is the link between the athlete/sportive amateur’s gut microbiota and TMAO production. It is well known that physical activity influences the population of gut microorganisms in a positive way, but it is quite evident that TMAO levels are also increased after exercise and sports [57]. Maybe this is a consequence of an omnivorous diet or one medium/high in animal protein that most athletes and sportive amateurs follow. The use of some ergogenic types of supplements such as L-carnitine [18] or choline isoforms (e.g., choline bitartrate) [58] may also contribute to high TMAO levels. However, TMAOs levels are not high in individuals on lacto-ovo vegetarian diet (egg, yolks and whole dairy products included) or vegan diet [30]. Nowadays, many professional and amateur athletes are switching to vegetarian and vegan diets, which could be a reason why TMAO plasma levels are low in these subjects [59]. Most of the caloric intake of athletes comes from carbohydrates, mainly starchy foods, such as whole grain cereal and tubers, but even fruits rich in fibers and oligosaccharides [60] that influence the gut microbiota by increasing *Bacteroidetes* phylum such as *Prevotellaceae* family and *Actinobacteria*. These families are partially responsible of TMA derived compounds conversion, but more importantly, they produce short-chain fatty acids (SCFA) [47]. In some studies, *Firmicutes* appears to be high in athletes and it is the main phylum implicated in TMA conversion [34,46,61]. Wolyniec et al. investigated TMAO levels before and after amateur runners’ 10 km or 100 Km ultramarathon there was no significant change in the levels, but only an acute increment. The only significant change was a 3.9-fold increase of TMAOs in the fastest runners of the 100 km race. The authors speculated that TMAOs level may impair the runner’s performance [62]. Certainly, high intensity physical activity requires a massive turnover of enzymes and substrates, leading to a huge production of metabolites, e.g., many L-carnitine isotypes that may influence TMA and TMAO conversion [63]. The athlete/sportive amateurs’ gut microbiota frequently shows some controversial aspects. Some studies report claims that physical activity reduces *Firmicutes* but increases *Bacteroidetes*, possibly due to the influence of the diet rich in whole grain carbohydrates, fruits, and vegetables [53,64]; others report the opposite effects because *Firmicutes* seems to be high in high caloric intake as most of athletes do [65]. It is undeniably controversial that some extreme physical activity shows an increment of TMAO levels, which already know that are strongly associated with CVD, obesity and type II diabetes, low-grade inflammation, gastrointestinal cancer [42], but at the same time reduce all these pathologies [66,67,68,69,70,71].

## 3. TMAO in Pathological Conditions

### 3.1. TMAO in Atherosclerosis and Cardiovascular Disease

Numerous studies indicate that gut microbiota is involved in the pathogenesis and progression of various cardiovascular diseases (CVD), such as heart failure (HF). HF causes changes in the composition of the intestinal microflora, which may affect the circulating levels of TMAO in human body. Researchers suggested intestinal strains, from *Firmicutes* and *Proteobacteria* phila, which can produce TMA, such as: *Anaerococcus hydrogenalis*, *Clostridium asparagiforme*, *Clostridium hathewayi*, *Clostridium sporogenes*, *Escherichia fergusonii*, *Proteus penneri*, *Providencia rettgeri*, and *Edwardsiella tarda*. The strains of these bacteria show an increased proportion in patients with HF. This indicates that changes in intestinal microbiota may affect TMAO levels by regulating TMA synthesis in the intestines [72]. Direct and indirect active roles of TMAOs in atherosclerosis and CVD are well established [5,30,39,42], also their associations in obesity and diabetes mellitus [19,29,39,73,74] as well as in low-grade inflammation [3,75] that are often are comorbidities. All of these may eventually also develop cancer [1,33,76,77,78]. The high levels of circulating TMAO in blood are strongly correlated to cardiovascular events such as stroke, myocardial infarctions, peripheral artery disease, acute coronary syndrome, and atherosclerosis [38,48,79,80]. Indeed, TMAO levels are correlated with the size of aortic atherosclerotic plaque, and they play a pivotal role as a pro-atherogenic factor. TMAO participates actively in the early stage of atherosclerotic process by promoting the macrophages migration, contributing to foam cell formation in the arterial intima [30,81]. Furthermore, high TMAO levels lead to an accumulation of ox-LDL particles within the macrophages by upregulating CD36 and scavenger receptor SR-A1 (Scavenger Receptor A1). These scavenger receptors are responsible for the transformation of macrophages into foam cells [82,83,84,85]. At the same time, the high levels of TMAO increase the expression of inflammatory cytokines, such as TNF-alpha and IL-6, that promote the migration of macrophages and their accumulation in arterial intima [82,86]. TMAO molecules seem to be involved even in endothelial dysfunction, a prelude to cardiovascular disease [7,36,81,87]. Vascular endothelial damage was observed in mice fed with a choline-rich diet [88], and high TMAO levels were associated with an increased systemic inflammation, oxidative stress, and fewer circulating endothelial progenitor cells (EPCs) [89,90,91,92,93] EPCs with a “spindle-shape” morphology, can take up acetylated LDL particles (acLDL) [94], whereas high TMAO levels reduce this. The formation of AcLDL in healthy subjects remains controversial, but it is often used as a model of oxidized or glycated LDL taken up by scavenger receptors. The potential accumulation of oxidized LDL particles is a precursor of inflammation and CVD [95]. TMAO can directly and indirectly< activate inflammatory signals such as NF-kB in aortic endothelial cells, and thereby contribute to atherosclerogenesis [96]. Furthermore, TMAOs reduce the expression of anti-inflammatory cytokines such as IL-10 that can protect the endothelial tissue from damage and inflammation, block the activity of NF-kB [97], and inhibit the adhesion of monocytes on the endothelial cells by downregulating the expression of CD18 and CD62-L on immune competent cells [98]. Finally, TMAOs can increase oxidative stress and reduce the endothelial nitric oxide synthases (eNOS) [99]. Oxidative stress is one of the most powerful promoters of atherosclerosis [87,96], whereas eNOS is a protective factor of endothelial health [100,101,102]. TMAO may have direct or indirect effects on (i) promotion macrophages migration and foam cells forming in the arterial intima; (ii) the accumulation of ox-LDL in situ within the macrophages; (iii) the reduction of EPCs, IL-10, eNOS, with the consequent rise of NF-kB, oxidative stress, and the LDL particles level in blood. All these effects lead to endothelial damages and cardiovascular dysfunction [103].

### 3.2. TMAO in Chronic Kidney Disease

High TMAO levels were found in patients with a chronic kidney disease (CKD). In patients with CKD treated with hemodialysis, pre-dialysis TMAO plasma levels were 77 ± 26 µM/dL, whereas in control group 2 ± 1 µM/dL, and other studies confirm that TMAO plasma levels of CKD patients are up to 40 times greater than normal [7,73,104]. CKD is also associated with CVD or a low-grade inflammation. All these show high levels of circulating TMAOs [20]. Missailidis et al. suggest that after a renal transplantation, the levels of TMAO were normalized [105], indicating not only a crucial role of the kidney in the excretion of these molecules, but also in their production. Indeed, some authors report a strong link between TMAO circulating levels, events of CVD and an effect on kidney health [105,106,107], with a direct connection with a low renal function [42], because there is a transporter called organic cation transporter 2 (OCT2) in basolateral kidney membrane that is responsible for the uptake of TMAOs [108]. A recent revision of Dongsheng et al. [109] confirms that the mechanism by which TMAO may enhance renal damage and aggravate nephropathy has not been well established. High TMAO plasma and urine levels may have a negative impact on CKD, due to the activation genes expression in the kidney tissue, but the mechanism is not known yet.

### 3.3. TMAO and Type II Diabetes

Several studies associate high plasma levels of TMAO and T2D or prediabetes [19,29,39,73,74,110,111,112,113], but none has proven a direct cause–effect. In mice feed with high fat diets plus 0.2% of TMAO molecules, it seems to impair glucose tolerance by affecting gene expression on insulin pathways in the liver and by increasing mRNA levels of pro-inflammatory cytokines in adipose tissue [114]. A bidirectional Mendelian assessment reports that high TMAO levels in T2D patients is more like a consequence [115]. For now, we only conclude that the overall microbiota disproportion found in obese patients with pre-diabetes or T2D can lead to high plasma levels of TMAO [74,110,111,112,113].

### 3.4. TMAO, Microbiota Homeostasis and Cancer

The latest literature proved how the gut microbiota plays an important role in gastrointestinal cancer (GIC) [116]. Nowadays, the human microbiota is considered an organ that communicates in synergy with the other apparatus and with many physiological influences. Therefore, its homeostasis, called *eubiosis*, must be protected. When the microbiota is compromised, or when the proportions of microorganisms in it are modified, a syndrome called dysbiosis occurs [116]. Approximately 90% of these microorganisms constituting the microbiota belong to *Firmicutes*, *Bacteroidetes*, *Proteobacteria* and *Actinobacteria*. Viruses, eukarya, fungi, blastocystis, amoebozoa and archea [117,118,119,120,121] represent the other components. A good and healthy microbiota is considered necessary to regulate the immune function, intestinal mucosal protection, vitamins production, correct digestion, and nutrients absorption [122]. The most important phyla correlating with a healthy gut and, consequently, overall health, are the *Firmicutes* and *Cytophaga*-*Flavobacterium*-*Bacteroides* (CFB) that are indeed from *Bacteroidetes* phyla [118]. Dysbiosis is not only a difference of the microorganisms’ proportion, but it is correlated with various pathologies with different nature such as being overweight and severe obesity, which may carry over even to cardiovascular events, insulin resistance and type II diabetes [74]. A microbiota disproportion was founded and confirmed in some mental pathologies [123,124], such as depression, that it is important to include in the context of other pathologies which affect the digestive apparatus, such as inflammatory bowel disease (IBD) [125], liver disease [126], leaky gut and the intestinal mucosal function [127,128], and gastrointestinal cancer [116,129,130]. Gastrointestinal cancer is one of the most common neoplasia all over the world [131], and lot of data show how the microbiota interacts with this pathology [116,132,133,134,135,136]. Obesity and low-grade inflammation are two main factors which can lead to cancer development [75], and there is a strong correlation within obesity, low-grade inflammation, dysbiosis and colorectal cancer (CRC) [129,132,136,137].

In patients with gastrointestinal cancers, the most abundant family of microorganisms are the *Enterobacteriaceae* that are situated in the small intestine. A low presence of *Lactobacillaceae* and *Acidoaminococcaceae* is typical of colon cancers and *Bifidobacteriaceae* in those of the rectum [138]. At the species level, *Bacteroides fragilis* seem to account for many colorectal cancers and *Helicobacter pylori* for gastric cancers [116,129,139,140]. Even *Escherichia coli* [141] and *Enterococcus faecalis* [142] may be involved in cancer development by DNA mutation genes. Some studies have shown that chemotherapy in patients with GIC leads to a modification/restoration of microbiota dysbiosis [143], a much greater richness in *Lactobacillaceae* has been found after therapy, compared to untreated controls [138]. Another genus correlated with CLC, *Fusobacterium*, showed a significative reduction after debulking surgery, but not chemotherapy [144]. At last, in a very recent study that compared the microbiota composition in obese patients with CLC, non-obese patients with CLC and a healthy control, shows enormous differences in diversity and richness of the gut species [145]. A reduction in richness was found in both CLC groups in comparison with the healthy group, but much more incisive was the decrease in diversity. The major phyla detected in healthy group was *Bacteroidetes*, with more than 50%, whereas it was below 30% in the CLC groups. *Firmicutes* was 39–43% in CLC groups and about 21% in the healthy one, and *Fusobacterium* was 9.1% and 1.2%, respectively. In conclusion, there is strong evidence for a reduction of species which produce SCFA (*Butyricimonas*, *Roseburia*, *Blautia*, *Faecalibacterium*, and *Ruminococcus*), and an increase of pathogenic and induced-cancer ones (*Fusobacterium*, *Clostridium*, *Prevotella*, *Desulfovibrio*, and *Enterococcus*) [145]. In this context, the TMAOs molecules may be implicated too. An increased level in TMAO concentration may be caused by diet, changes in the composition of intestinal microflora, gut dysbiosis or impairment of the gut–blood barrier. Studies on mice have shown that intestinal bacteria are essential to convert dietary compounds to TMA [146]. The production of TMA and TMAO can be almost completely suppressed using broad spectrum antibiotics, and after one month of withdrawal of antibiotics, the TMAO concentration returns to normal [147]. Indeed, some studies have associated the high levels of TMAO with high TNF-alpha, IL-6, C-reactive protein [44], pro-inflammatory cytokines, IL-1beta [96] and even as a coadjuvant of *Helicobacter pylori* to promote infection in the gastric epithelial cells, increasing the activity of IL-6 and chemokine ligands. This suggests a potential link between TMAO and gastric cancer via the inflammatory process. Consistent with this, Yue et al. also demonstrated that TMAO can trigger the activation of the nod-like receptor family pyrin domain containing 3 (NLRP3) inflammasome [148,149], which was suggested to be implicated in the growth and/or metastasis of a variety of cancers including head and neck cancer, oral cancer, lung cancer, prostate cancer, and colorectal cancer [150,151]. Association studies provided further support for a link between TMAO and inflammation. The serum level of TMAO was shown to be positively correlated with the level of certain pro-inflammatory mediators including tumor necrosis factor-alpha (TNF-α) and IL-6. Moreover, studies have also displayed that an enhanced level of TMAO prompted the initiation of NF-Kappa-B route and improved the expression of pro-inflammatory genes involving chemokines, adhesion molecules and inflammatory cytokines [152]. Several data show, indeed, how the TMAO levels are quite high in patients with colorectal cancer and even higher in obese subjects with the same type of cancer [145,153,154]. Moreover, the presence of high quantity of *Firmicutes phylum*, *Prevotellaceae*, *Enterobacteraceae* and *Desulfovibrio* that can increase the conversion of choline to TMA by the expression of the cutC gene [34], and low levels of *Bacteroidetes* phylum [145] in relation to healthy controls with neither cancer nor obesity, are very illuminating and confirm the role of TMAO. This would indicate reducing all foods containing choline, betaine, lecithin, gamma-butyrobetaine, ergothioneine and L-carnitine, to avoid a massive conversion to TMAO [1]. Conversion of these compounds in TMA not only depends on the food sources [4,24,29], but also on microbiota composition [42] which is compromised by pathological status [129,137,155]. It is now clear that many gastro-intestinal pathologies are related to bacteria families of *Firmicutes* phylum [49], which even includes healthy strains of gut microbiota [50,51]. TMAO is frequently used as a risk marker of diseases, but its usefulness as a standalone marker is limited due to the high intra individual variability that may reflect dietary changes from day to day, in particular the intake of meat or fish [156]. Oxidative stress might also be one of the factors linking TMAO and cancer. Recent studies showed that TMAO could be implicated in oxidative stress and increased circulating TMAO was shown to induce superoxide production, a reactive oxygen species (ROS) linked to oxidative stress. In an in vitro assay, TMAO was also shown to stimulate the production of ROS in cells [157,158,159].

## 4. Practical Recommendations for Prevention and Treatments Reducing TMAO Production

### 4.1. Diet

To modulate the TMA and TMAO levels, the manipulation of food intake and diet is probably the best/most promising initial treatment. The targets are primarily choline, betaine, lecithin, gamma-butyrobetaine, ergothioneine and L-carnitine [3], naturally found in animal protein, processed and unprocessed meat, egg yolk, dairy products, and even fatty fish [26]. However, some of these compounds are essential nutrients, which eventually may necessitate supplementation, if their intake is inadequate. High fat, high protein or Western diets contain/are associated with high levels of TMAO [3], even when supplemented with fish oil [160]. In contrast, inclusion of some nuts such as pistachios [161] or some indigestible fiber [162], or vegetarian diets [30], tended to attenuate TMAO production.

Choline is a component of choline phospholipids, which are essential components of cell membranes [163,164]; it is also a precursor of acetylcholine, which acts as neurotransmitter [165] and plays a pivotal role in the correct development of brain cells such as astrocytes [166]. Furthermore, its presence is required in other tissues to interact with hormones, growth factors and neural cells [167]. Although it can be produced endogenously, dietary supplementation is necessary in certain conditions, such as vegan diets, pregnancy, diets very low in protein sources, or parenteral nutrition [163,164,168,169,170]. The recommended choline intake is 7 mg/kg/day for adults, 450 mg/day for women throughout pregnancy, and up to 550 mg/day to support breastfeeding [171]. An inadequate intake of choline and betaine is linked to pro-atherogenic changes [172,173]. For example, betaine is required to homocysteine methylation to methionine, and its insufficiency leads to a high circulating level of homocysteine [174], which is correlated, with CVD [175], cancers [176], neurodegenerative disease [177] and osteoporosis [178]. There is an interesting hypothesis of choline/1-carbon (betaine) crosstalk metabolism, in which low intake of choline and betaine may interact with several mitochondrial pathways having an impact on the systemic insulin sensitivity of muscle and adipose tissues, impairing the body composition, energy homeostasis and thus the health status [179]. A “sweet pot” daily dose of choline and betaine seems to be associated with better body composition [180]. The choline food sources are egg yolk, whole milk and whole dairy products, beef, pork, liver, seafood and fatty fish [9], whereas for betaine whole grains, shellfish beets and spinach are the common sources [181]. Apart from the hypothesized crosstalk with choline, betaine itself is another essential nutrient that can be obtained from veggies, shellfish, sugar beet and cereal [27] but is also produced from oxidation of choline in betaine aldehyde by choline dehydrogenase, and betaine aldehyde is converted in betaine by the betaine aldehyde dehydrogenase [182]. The molecule is also known as trimethyl-glycine for its 3 methyl groups; its function is to protect cells from oxidative stress and to add a methyl group, as aforementioned, during the conversion of homocysteine in methionine [181], an important process to low levels of homocysteine in the blood that is a supposed CVD marker [175]. It is established that choline and betaine are precursors of TMA and eventually TMAO, but it is also certain that they are essential nutrients, so it is suggested to cover, but not to exceed the Recommended Dietary Allowance (RDA) of choline. For the betaine, association studies in some populations suggest that its intake is usually half that of choline [180]. The choline and betaine content in food is shown in Table 1. The risks of conversion of choline in TMA by the bacteria *Desulfovibrio* cutC gene expression [34] is tangible, but only choline bitartrate and not phosphatidylcholine seems to raise the TMAO levels and excretion with urine [183] in those who are considered “high TMAO producers” with abundance in microbiota of *Firmicutes* phylum, *Clostridia* class such as *Clostridium*, *Ruminococcaceae* and *Lachnospiraceae* [184]. Since egg yolks contain mostly phosphatidylcholine, 2–3 whole eggs/day (roughly 400 mg choline) result in high levels of choline but not TMAOs in the bloodstream [185,186].

L-carnitine is an amino acid that is essential for the organism at mitochondrial level to transport long chain fatty acids into the matrix to produce energy via beta-oxidation [187]. Supplementation is not required to maintain a physiological level, because the body can produce the right amount endogenously from lysine and methionine [187] and the kidneys reduce or increase the excretion based on current blood levels [187,188]. L-carnitine is easily converted into TMA in gut microbiota by the 2-component Rieske-type L-carnitine oxygenated CntA/B in the presence of oxygen molecule [11] and is strongly correlated with an increase of TMAO levels [12,30]. L-carnitine is contained mostly in red meat and for a very small part in white meat (poultry) [189]. A diet rich in red meat results in a high level of L-carnitine and gamma-butyrobetaine and consequently in high TMAO levels [1,11,18,21,23,24,25,30,44,189,190]. Vegetarians, who include in their diet whole dairy products and whole eggs are less predisposed to convert L-carnitine into TMA, even with a supplementations protocol [30]. This may be due to their microbiota composition [30] or their low levels of L-carnitine from the meatless diet. Since L-carnitine supplementation shows no benefits at all, with an exception for those who have a specific deficiency [18], the most recent international guidelines against cancers [191] recommend the entire population, for patients with CVD, type II diabetes, CKD, and cancer, to avoid supplements of any form of carnitine and to limit all foods containing it [1]. At last, a diet very rich in fat, a normal Western diet or a diet mainly composed of protein and fat with low or no sources of carbohydrates and fibers (e.g., ketogenic diet, very low carb diet) leads to an acute postprandial and chronic production of TMAOs, by changing the ratio between *Firmicutes* and *Bacteroidetes* in favor of the former [3]. Poor quality diets also create dysbiosis [122]. The classical beneficial Mediterranean diet does not lower TMAOs levels after six months of intervention, maybe because of the fish intake that directly brings free TMAOs molecules into the organism. Indeed, as Griffin et al. suggest, it would probably be useful to investigate whether the Mediterranean or another adequate healthy diet along the anti-cancer international guidelines, could affect TMAOs serum levels with or without fish [192].

### 4.2. Drugs, Supplements and Physical Activity

An adequate diet rich in whole grains, natural starchy foods, fruits, and vegetables, containing moderated choline sources and low to no L-carnitine would seem to be the first step to reduce circulating TMAOs by directly decreasing those compounds that convert to TMA. Probiotics and prebiotics could be used to modify microbiota targeting TMAOs. Indeed, the administration of probiotic *Lacticaseibacillus paracasei* [193], but not *Lacticaseibacillus casei* [194] or *Lactiplantibacillus plantarum* [195] have demonstrated to reduce circulating TMA and TMAO levels; *Enterobacter aerogenes* ZDY01 increased *Bacteroidales* (*Bacterodetes* phylum) and decreased *Prevotellaceae* and *Helicobacteraceae* families [196], and supplementing with *Archeobacteria* phylum, which subtract methyl compounds required to form TMA and decrease in TMAO [17,197]. In contrast, prebiotic supplementing with *Arabinoxylan* oligosaccharide plus vitamins B and D showed only a little reduction in serum TMAO [198]. Using a prebiotic such as resveratrol, *Bacterodetes* phylum is increased, while *Firmicutes*, aside *Lactobacillus* and *Bifidobacterium* genus, decreased along with TMAO plasma levels [199,200]. Therefore, a prudent approach would be to evaluate probiotics and prebiotics as coadjuvant therapy with diet on TMAOs modulation. Physical activity also has an important effect on the composition of gut microbiota [201,202]. Endurance training, for example, lowers the *Proteobacteria* and enhances *Akkermansia muciniphilia* [203], decreases *Clostridium difficile*, increases *Oscillospira* [204] and augments beneficial short-chain fatty acids in lean people [205]. As the intensity and volume of physical activity increases, such as in professional athletes or the military, the positive effects on gut microbiota becomes inversely proportional [206,207], with an increment of potentially pathogenic *Staphylococcus*, *Peptostreptococcus*, *Peptoniphilus*, *Acidaminococcus* and *Fusobacterium*, and a reduction of potentially beneficial strains [208]. However, prebiotics and probiotics are/seem indicated in athletes with compromised immune function, upper respiratory tract illnesses (URTIs), or gastrointestinal disorders such as diarrhea, bloating, abdominal pain or gastroesophageal reflux, all of which strongly correlate with dysbiosis [206,209]. Even though physical activity and sports in general does not seem to affect the gut microbiota and TMA conversion, they remain correlated to an overall beneficial impact on various conditions that favor gastro-intestinal cancer development [66,68,69,71,206]. A highlight could be put on the *Akkermansia muciniphilia*, which is increased by physical activity [202,203], administration of metformin [210,211] and berberine [212,213,214,215,216]. *Akkermansia muciniphilia* appears to be a promising strain that can be beneficial in gastro-intestinal cancer and in the management of glucose in obese patients [50,132,217,218].

Antibiotics have a great impact on the gut microbiota. They are the most incisive drug that can block TMAOs production, but at the same time, they kill other beneficial microorganisms.

Ciprofloxacin and metronidazole are the most effective suppressors of TMAOs, but after just one month of use, TMAO levels rise again [48]. Even a mix of various broad-spectrum antibiotics such as vancomycin, neomycin-sulphate, metronidazole and ampicillin block the conversion of choline into TMA, and therefore reduce TMAO, but result in relatively fast development of antibiotic-resistance and extinction of other beneficial phyla [6,48].

The compound 3, 3-Dimethyldimethyl-1-butanol (DMB), a natural compound derived from vinegar, olive, and grapeseed oil, could be used to limit the conversion of choline, betaine and L-carnitine to TMA with imbibition of TMA lyase but unfortunately not of gamma-butyrobetaine (GBB) to TMA and neither the FMO3 conversion to TMAO [219]. Other second generation of choline analogues are Flouro-methylcholine and Iodo-methylcholine, which irreversibly block TMA lyase, showed a decrease of TMAOs and thrombotic events too without any toxic effects [220]. 

Meldonium, another molecule used in ischemic and atherosclerosis events, decreases TMAO levels by blocking the conversion from L-carnitine to TMAO and GBB to L-carnitine [221]. In fact, it leads to an accumulation of GBBs and does not have any effects on choline conversion to TMA [222]. 

Enalapril is an ACE inhibitor drug that lowers TMAO levels. It increases the excretion of TMAO, possibly by a common sodium mechanism, but does not reduce TMA levels [223]. 

A study of 2 g/day metformin without diet control did not show an effect on TMAO levels, even though metformin affects the microbiota [224]. In contrast, a very recent study reported that 250 mg/kg metformin for 4–8 weeks lowered TMA and TMAO levels in db/db mice that was treated only metformin and with metformin plus a choline bolus to mimic a typical Western diet [225]. This study also evaluated the effects of metformin on the choline-TMA lyase genes expression (CutD, CutC, cmcA) and FMO3 liver enzymes, and found that metformin had an impact on gut microbiota species that decrease the conversion of substrates to TMA resulting in a decrease of TMAOs [225].

Finally, one study reported that 81 mg aspirin plus choline diet (two whole eggs a day) significantly reduced TMAO plasma levels over 12 months [107,226,227].

## 5. Conclusions

**Figure 1:**
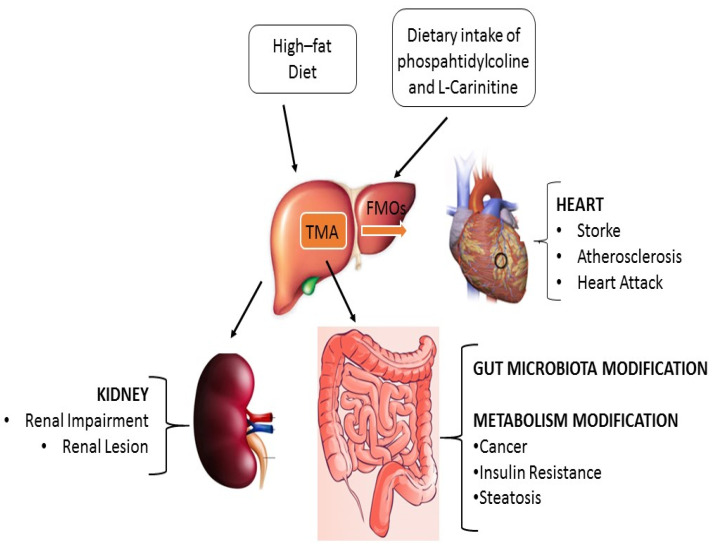
The action of TMA in pathological condition. The dietary intake of choline isoforms, carnitine, gamma-butyrobetaine contribute to increment the plasma level of TMA. TMA are converted in TMAO in the liver. TMAO are involved and correlated with kidney and liver disease, gastrointestinal cancers, diabetes type II, atherosclerosis, and cardiac muscle damage.

Different studies have highlighted how the microbiota can be a direct or indirect cause of the onset or progression of various types of pathologies. Foods, or nutrient molecules extracted from the diet, can be powerful modulators of the microbiota. One of these, TMAO, is formed from the precursor of TMA (trimethylamine) through the combined action of the intestinal microbiota and the liver, as we can see in the Figure 1. TMAO directly interferes with hepatic gluconeogenesis and glucose transport, increasing the susceptibility to insulin resistance. A high level of TMAO has been associated with an increased risk of adverse events in cardiovascular disease. Furthermore, TMAO can cause epigenetic changes to DNA and, through the formation of N-nitrous, compounds damage DNA, which can lead to malignant transformations in exposed cells. Before blaming some natural foods, that contain important and useful compounds for our health, it would be right and proper to understand how the intestinal microbiota, individual polymorphism and lifestyle of a subject can modulate the expression of TMAO. Several therapeutic strategies are being studied to reduce TMAO levels, including the use of broad-spectrum oral antibiotics, promoting the growth of bacteria that use TMAO as a substrate and the development of target-specific molecules. Despite the accumulated evidence, one wonders whether TMAO is a spectator’s mediator in the disease process. Therefore, it is important to undertake studies to establish the role of TMAO in human health and disease. Recent investigations into the cross-dialogue between GM and human health have opened new approaches for diet-based interventions. Manipulation of gut microbiota through diet is a visionary approach to improve human health. On the bases of this knowledge, we propose that the administration of specific foods could be a new support in daily practice, as shown in Table 2. Based on the current range of carnitine intake (i.e., from 2 to 12 mol/kg/day or from 22.7 to 136.1 mg/day for a 70 kg human being), a standard diet provides enough carnitine, 3.4 times the lower level or 96% of the average recommendation. However, a detailed characterization of both the extent and the mechanisms by which these interactions occur will be necessary. The principle of primary prevention of oncological and cardiovascular diseases is certainly based on the dietary modifications and healthy behaviors. The intake of a specific food category, and the combination with the genetic, epigenetic and characteristics of the microbiota of a single subject has to be considered more frequently in future precision medicine.

## Figures and Tables

**Table 1:** Betaine, phosphocholine and free choline raw food contents in g for 100 g [1].

	Betaine	Free Choline	Phosphocholine
Egg yolk	0.0	2.38	5.84
*Milk*			
Whole 3.25% Fat	0.6	3.7	1.8
2% Fat	0.9	2.8	1.6
*Butter*	0.3	0.5	0.7
*Cheese*			
Cheddar	0.7	1.6	0.6
Cottage 2%	0.6	2.9	1.3
Mozzarella	0.7	2.3	0.9
Swiss	0.6	4.5	0.0
*Chicken*			
Meat and skin	7.8	6.0	3.6
Liver	16.9	49.2	4.1
*Pork*			
Sausage	3.4	8.0	0.5
Bacon	0.9	4.4	1.4
Loin, lean only	2.4	1.6	2.2
*Beef*			
Ground 80% lean	8.2	2.6	0.4
Liver	4.4	56.2	11.8
*Fish and seafoods (cooked*)			
Cod (Altantic)	9.7	17.7	1.6
Shrimp	33.0	1.5	0.8
Salmon (Sockeye)	2.1	8.6	1.1
Tilapia	25.3	21.4	2.5
Tuna (canned in water)	2.7	2.1	0.0
*Vegetables*			
Beets	128.7	4.1	0.9
Broccoli	0.1	18.1	0.4
Cabbage	0.4	6.1	2.3
Carrots	0.4	6.8	1.1
Lettuce iceberg	0.1	4.8	1.5
Mushrooms	10.7	5.9	1.3
Potato, white, flesh and skin	0.2	7.9	0.3
Spinach boiled, drained	726	1.7	1.1
Tomato paste	0.4	26.2	4.3
Tomato	0.1	4.4	1.8
*Nuts (roasted and dried)*			
Almonds	0.5	9.4	1.9
Brazilnuts	0.4	16.1	0.3
Cashews	11.2	19.6	0.9
Hazelnuts	0.4	15.2	0.9
Macadamia	0.3	11.3	1.0
Pecans	0.7	9.7	1.3
Pine	0.4	8.4	2.1
Pistachio	0.8	10.7	8.5
walnuts	0.5	8.3	0.5
*Legumes*			
Beans, kidney, canned	0.1	19.7	0.5
Peanut butter, smooth	0.4	25.8	0.7
Soy milk	0.8	13.1	3.4
Soy sauce (shoyu)	39.6	31.0	0.0
Cereal grains and others			
Oat bran	35.7	4.4	0.7
Rice, brown	0.5	4.7	0.0
Pasta, dry	460	9.7	0.0
Wheat flour, white	124.4	5.7	0.1
Bread, wheat	85.2	11.5	0.3
Kellogg’s all-bran	360.0	25.5	1.7

**Table 2:** L-carnitine raw food contents in mg for 100 g [2].

*Beef*	
Steak	65.0
Ground	87.5
Tenderloin	78.6
T-Bone	84.2
Loin	64.6
*Chicken*	
Liver	94.0
Meat	10.4
Wing Meat	10.0
*Turkey meat*	21.2
*Lamb Chop*	40.5
*Pork*	
Shoulder	21.1
Ham	53.5
White Ham	33.5
Sausage	7.1
*Veal*	
Shoulder	78.2
Sirloin	132.8
*Milk*	
2% fat	2.9
4% fat	2.3
*Butter*	1.3
*Cheese*	
Camembert	14.4
Gruyere	6.5
Feta	1.8
Goat cheese	15.3
Mozzarella	0.3
Parmesan	0.7
*Yogurt*	
Regular	12.2
0% fat	12.5
*Egg*	
White	0.3
Yolk	0.8
*Fish and seafood*	
Anchovy	1.8
Shrimp	0.7
Cod (Antlantic)	1.8
Hake (boiled)	2.9
Mussels (cooked)	2.6
Salmon (cooked)	5.8
Smoked salmon	1.0
Tuna	1.5

## Data Availability

Not applicable.
